# Intragenomic rDNA variation - the product of concerted evolution, mutation, or something in between?

**DOI:** 10.1038/s41437-023-00634-5

**Published:** 2023-07-04

**Authors:** Wencai Wang, Xianzhi Zhang, Sònia Garcia, Andrew R. Leitch, Aleš Kovařík

**Affiliations:** 1 Science and Technology Innovation Center, Guangzhou University of Chinese Medicine, Guangzhou, 510405 China; 2 Department of Horticulture, College of Horticulture and Landscape Architecture, Zhongkai University of Agriculture and Engineering, Guangzhou, 510225 China; 3 Institut Botànic de Barcelona, IBB (CSIC - Ajuntament de Barcelona), Barcelona, Spain; 4 School of Biological and Behavioral Sciences, Queen Mary University of London, London, E1 4NS UK; 5 Institute of Biophysics, Academy of Sciences of the Czech Republic, Brno, CZ–61200 Czech Republic

**Keywords:** Evolutionary genetics, Evolutionary biology, Plant evolution

## Abstract

The classical model of concerted evolution states that hundreds to thousands of ribosomal DNA (rDNA) units undergo homogenization, making the multiple copies of the individual units more uniform across the genome than would be expected given mutation frequencies and gene redundancy. While the universality of this over 50-year-old model has been confirmed in a range of organisms, advanced high throughput sequencing techniques have also revealed that rDNA homogenization in many organisms is partial and, in rare cases, even apparently failing. The potential underpinning processes leading to unexpected intragenomic variation have been discussed in a number of studies, but a comprehensive understanding remains to be determined. In this work, we summarize information on variation or polymorphisms in rDNAs across a wide range of taxa amongst animals, fungi, plants, and protists. We discuss the definition and description of concerted evolution and describe whether incomplete concerted evolution of rDNAs predominantly affects coding or non-coding regions of rDNA units and if it leads to the formation of pseudogenes or not. We also discuss the factors contributing to rDNA variation, such as interspecific hybridization, meiotic cycles, rDNA expression status, genome size, and the activity of effector genes involved in genetic recombination, epigenetic modifications, and DNA editing. Finally, we argue that a combination of approaches is needed to target genetic and epigenetic phenomena influencing incomplete concerted evolution, to give a comprehensive understanding of the evolution and functional consequences of intragenomic variation in rDNA.

## Introduction

**Fig. 1:**
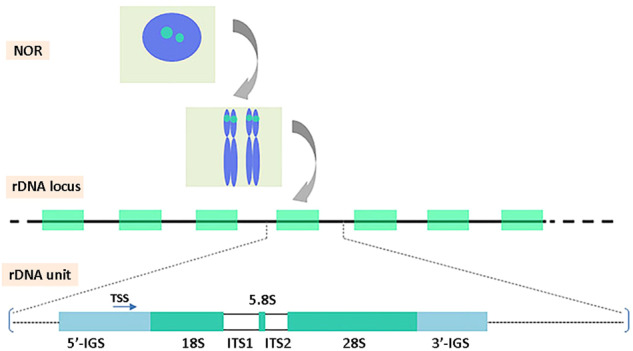
Scheme of 45S rDNA unit structure and their genomic organization. The rDNA (green dots) at the nucleolar organizer regions (NOR) at interphase and on metaphase chromosomes represent rDNA loci, with each rDNA locus comprised of arrays of rDNA units (green squares) separated by intergenic spacer sequences (IGS). Each unit is comprised of the 18S, 5.8S, and 28S rDNA subunits, separated by the internally transcribed spacers (ITS). TSS, transcription start site.

Ribosomal DNAs (rDNAs) are best known for being the most conserved and heavily utilized house-keeping genes, encoding in eukaryotic organisms four types of structural ribosomal RNAs (rRNAs), i.e. 5S, 5.8S, 18S, and 28S/26S/25S rRNA. Three of these (5.8S, 18S, and 28S/26S/25S) are derived, respectively, from 18S-5.8S-28S/26S/25S rDNA (in this order) that are always linked in a single unit known as 45S rDNA in animals and 35S rDNA in plants. By convention, the nomenclature of rRNA/rDNA is derived from sedimentation rates of rRNA macromolecules which differ between the groups (Hemleben et al. 2021). The 45S/35S rDNAs will hereafter be referred as “45S rDNA(s)” and the 28S/26S/25S rDNAs as “28S rDNA(s)” for reading convenience. In eukaryotes, rDNAs form a multi-copy family of sequences organized in tandem repeats across one or several loci. The individual genes are separated by internal transcribed (ITS1 and ITS2) and intergenic (IGS) spacers (Fig. 1). The active 45S rRNA genes constitute important chromosomal landmarks called nucleolar organizer regions (NORs). In mammals and in most seed plants 5S rDNA, coding 5S rRNA, is separated from 45S rDNA at independent loci. However, a physical linkage of 5S with 45S genes is found in some plant groups (Garcia et al. 2009; Garcia and Kovařík 2013) especially in early diverging plants (Sousa et al. 2020; Wicke et al. 2011), some vertebrates (Davidian et al. 2022) and invertebrates (Drouin et al. 1987, 1992) and is commonly encountered amongst yeasts (Petes 1980; Szostak and Wu 1980).

In eukaryotic genomes, there is amazing sequence similarity between rDNA units observed within the genomes, indicating that each unit does not evolve independently from others, i.e. their evolution is concerted (Brown et al. 1972; Zimmer et al. 1980). The high similarity of units, i.e. homogenization, has been explained by repeated unequal crossovers (Dover 1982) and modeled in computer simulations almost 50 years ago, where mutations arising in repeats are erased over generations (Smith 1974). However, similarity does not mean complete sequence identity and in many natural situations we witness variable levels of unit-to-unit variation. Such variation has been reported in a broad range of eukaryotes, including plants (Chelomina et al. 2016; Harpke and Peterson 2006; Mayol and Rosselló 2001; Osuna-Mascaró et al. 2022; Shao et al. 2018; Vazquez 2019; Wang et al. 2016; Weitemier et al. 2015; Xiao et al. 2010), mammals (Robicheau et al. 2017), fish (Pinhal et al. 2011), insects (Keller et al. 2006) and fungi (Sultanov and Hochwagen 2022; West et al. 2014) (for a more complete list, see Supplementary Table 1). In some genomes, mutations in coding regions render rDNA units inactive. For example, humans carry c. 300 copies of 45S rDNA (1 C genome), out of which 14 (5%) are pseudogenes harboring various mutations (Robicheau et al. 2017) and the number of pseudogenized copies of 5S rDNA is even higher (Sørensen and Frederiksen 1991). In the plant *Cycas revoluta*, it has been estimated that the fraction of 45S rDNA pseudogenes is even larger, reaching almost five thousand copies, c. 50% of the total number of rDNA copies (Wang et al. 2016). Sequence variation between rDNA units has been widely used to determine phylogenetic inference (Poczai and Hyvonen 2010). However, rDNA polymorphisms can provide both invalid phylogenetic relationships (Won and Renner 2005) and overestimation of species diversity (Sun et al. 2013). On the other hand, they can be utilized to map evolutionary histories in hybrid species (Rauscher et al. 2004; West et al. 2014) and resolve cryptic introgressants (Garcia et al. 2020).

Extensive studies have investigated and speculated upon the underpinning causes of intragenomic rDNA variations in a range of organisms (reviewed, e.g. in Smirnov et al. 2021; Symonová 2019), but much information remains to be collated and further developed, in particular a need for complete sequencing through rDNA loci. In this work, we initially summarize studies showing intragenomic polymorphisms in rDNA in animal, fungi, and plant species, occurring widely across much eukaryotic diversity. We then discuss biological factors influencing concerted evolution and contributing to rDNA diversity. Finally, intra-array diversity, i.e. nucleotide variation between the units within a single array, is proposed as a hallmark of inefficient or even failing concerted evolution.

## Intragenomic rDNA variations occur in divergent genera across the eukaryote tree-of-life

We collected publications by searching “intragenomic variation, non-concerted/incomplete concerted evolution of rDNA, rDNA polymorphism” in Google Scholar (up to 2022). This identified 136 records covering plants (32%), animals (36%), fungi (28%), protists (1%), and some prokaryotes (3%), and involving about 300 species in total (Supplementary Table 1). It is significant to note the following:

(i) Frequent intragenomic variations of 45S rDNA were found in ITS subregions (57% of all reports) (Supplementary Table 2). This might explain the occurrence of poor support for branch positions when using ITS markers in phylogenetic studies (Poczai and Hyvonen 2010). For instance, Song et al. (2012) investigated 178 plant species and found that intragenomic variation of ITS2 was frequent, with an average of 35 variants in each species’ genome. Exceptionally high intragenomic polymorphism in ITS was reported in *Mammillaria* (Harpke and Peterson 2006), *Asclepias* (Weitemier et al. 2015), and *Cycas* (Xiao et al. 2010). The extent of ITS intragenomic diversity may vary significantly among genera and even species within the same genus (Vazquez 2019; Weitemier et al. 2015).
(ii) Species with rDNA polymorphisms in plants, animals, and fungi (Supplementary Table 2) do not have close phylogenetic relationships, i.e., rDNA variations are scattered across eukaryote diversity and do not show apparent variation with genome size. Some genera had only a few variants (indicated as Single Nucleotide Polymorphisms, SNPs) in a few subregions, whereas other genera have extensive variation across the whole rDNA unit (Supplementary Table 1).
(iii) Variation in rDNA coding regions was generally several-fold lower than that of non-coding regions of rDNA units (Stage and Eickbush 2007). This is explained by the fact that ITS (and IGS) is under relaxed selection compared to the coding regions, the latter with high functional constraints and lacking third codon redundancy as is found in protein coding genes. However, both 18S and 28S rRNA genes contain subdomains, termed “core” and “expansion” regions (Hancock and Dover 1988), differing in the degree of sequence uniformity. Variation in expansion regions is more frequent than in cores (Stage and Eickbush 2007) which exhibit purifying selection signatures (Sultanov and Hochwagen 2022). Of note, differences between coding and non-coding regions were more obvious when using high frequency variant call cut-offs rather than low-frequency call cut-offs, underlining the importance of variant calling parameters in data interpretation.
(iv) The rDNA copy number can change rapidly over a few generations despite being similar, on average, between parents and offsprings (Rabanal et al. 2017 and reviwed in Kindelay and Maggert 2023; Salim and Gerton 2019). Indeed, shifts in copy number may significantly influence genome size such that it is visible to selection, as shown in the plant *Arabidopsis thaliana*, which has a relatively small genome (Long et al. 2013). In addition, the size of the array may influence non-ribosome-related functions of rDNA, such as the maintenance of genome integrity (Kobayashi 2008).
(v) Finally, variation between units is not limited to 45S rDNA, but it also occurs at 5S rDNA loci (Kellogg and Appels 1995; Schneeberger and Cullis 1992; Stepanenko et al. 2022; Tynkevich et al. 2022). In humans and mice, the copy number of 5S and 45S rDNA (occupying separate loci) seems to be harmonized between populations/strains (Gibbons et al. 2015) while at the genus level both loci tend to undergo independent evolution (Fehrer et al. 2021; Mahelka et al. 2013; Volkov et al. 2017) though both are likely to be influenced by similar genetic processes, namely amplification, recombination and elimination.

Certainly, there is no doubt of the occurrence of intra and intergenomic variations in rDNA unit copies. However, many observations of “rDNA diversity” need to be interpreted with caution. For example, the employment of different technical approaches used to identify rDNA variation may lead to results that are differentially interpreted, and it is difficult to compare the conclusions of these studies. For example, it is unclear to what extent different methodologies used to quantify polymorphisms or variations in rDNAs impact the results, e.g., it is not easy to compare results from RFLP (restriction fragment length polymorphism), PCR-cloning, high throughput sequencing (short and long reads) and the different bioinformatic methods used (e.g. SNP calling parameters). Moreover, artifacts stemming from PCR and amplification processes induce mutations or chimeras and the extent of biased amplification of pseudogenes is not known (Cronn et al. 2002). High throughput sequencing is also not without its problems. For example, rDNA units are inherently GC-rich, and contain microsatellites and other tandem repeats, particularly in the IGS. All these features negatively influence sequencing efficiency, resulting in low coverage of rDNA and even erratic base calls (Fan et al. 2022; Guiblet et al. 2018). Thus, ideally, a common methodology needs to be employed to accurately assess and compare the efficiency of concerted evolution in individual species.

## Factors leading to incomplete concerted evolution of rDNAs

**Fig. 2:**
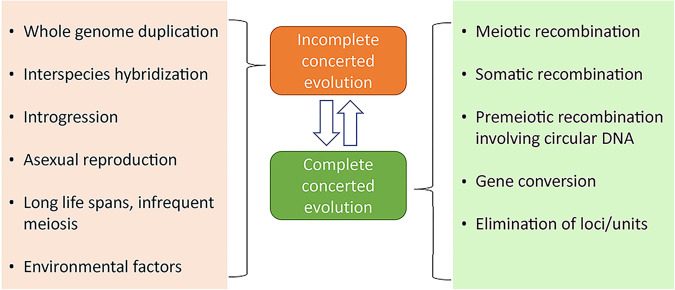
Biological processes leading to complete and incomplete concerted evolution phenotypes. Left panel: Processes increasing the heterogeneity of rDNA arrays. Right panel: Processes decreasing their heterogeneity.

The incomplete concerted evolution of multigenic families results from inefficient homogenization processes (Eickbush and Eickbush 2007; Liao 1999). Here we discuss possible biological processes (Fig. 2) that influence homogenization within rDNA.

### Interspecific hybridization and whole genome duplication

Interspecific hybridization linked with subsequent whole genome duplication (WGD), termed allopolyploidy, immediately results in rDNA heterogeneity since parental genomes contain different rDNA variants. Persistence of parental variation generates intragenomic heterogeneity and “incomplete concerted evolution” phenotypes. Evidence for this comes from a range of studies. For example, in *Saccharomyces cerevisiae*, hybrid strains have mosaic-like genomes and, on average, a nearly 3-fold higher rDNA variation than non-hybrid strains with “clean” structured genomes (James et al. 2009). In the plant *Malus toringoides*, a hybrid descendent of *M. transitoria* and *M. kansuensis*, ITS sequences of both parents are retained (Liang et al. 2015). We should bear in mind that the timing and extent of genomic changes following hybridization and WGD events vary between species and that these can affect the extent of rDNA homogenization. Some genomic alternations take place immediately with the onset of genome merger or WGD, whereas others take many generations (Adams and Wendel 2005). For example, only two generations were needed to homogenize rDNA in *Armeria* hybrids (Fuertes Aguilar et al. 1999). Also, significant differences exist between allopolyploid populations of independent origin (Borowska-Zuchowska and Hasterok 2017; Kovarik et al. 2005; Lowe and Abbott 1996; Sochorova et al. 2016) and even between individuals of the same origin (Bao et al. 2010) suggesting that changes at rDNA loci may be astonishingly fast upon ‘genomic shock’ induced by interspecific hybridization. However, intragenomic variation in IGS seems to be never entirely removed, possibly because of the inherent instability of the elements in its sequence structure (Lunerova et al. 2017). The adaptive significance, if any, of variation in IGS is debatable, since except for promoters and splicing site regions, the function of IGS is mostly unknown or absent (Fedoroff 1979).

WGD generates a large rRNA gene dosage change in a newly formed polyploid. It can be hypothesized that rDNA loci are particularly sensitive to WGD since the number of active genes needs to be harmonized with cellular requirements and organism physiology. In support of this hypothesis, the physical elimination of rDNA loci (both 5S and 45S) following WGD is commonly encountered and well-documented in multiple animals (Gromicho et al. 2006; Knytl et al. 2023; Roco et al. 2021; Symonová et al. 2017a; Tagliavini et al. 1999; Ye et al. 2017) and plant (Garcia et al. 2017; Kotseruba et al. 2003; Lim et al. 2007; Volkov et al.^ 2007^) polyploid systems. Significantly, in some cases, uniparental elimination of loci has occurred even in synthetic polyploid lineages (Guo and Han 2014; He et al. 2012; Malinska et al. 2010; Pontes et al. 2004). Such locus loss is actually reducing intragenomic rDNA heterogeneity following allopolyploidy and could potentially have adaptive significance.

**Fig. 3:**
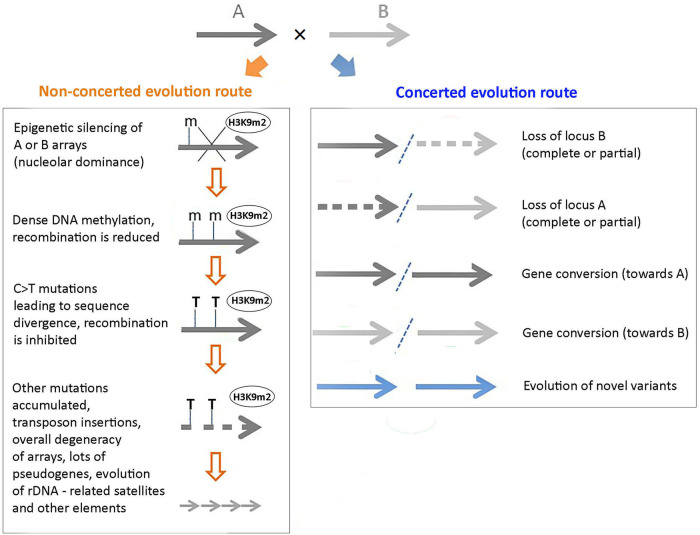
Hypothetical model showing complex evolutionary trajectories of rDNA in hybrids and allotetraploids. Top: parental A and B arrays (thick horizontal arrows) are unified in a newly formed nucleus after a hybridization event. The arrays take either a non-concerted evolution route leading to decreased homogeneity and incomplete homogenization phenotypes (left) or a concerted evolution route leading to increased homogeneity and complete homogenization phenotypes (right). In the left panel, only array A is shown for simplicity. Heterochromatic marks such as dimethylation of histone H3 lysine 9 (H3K9m2) (ovals in left) and cytosine methylation (“m” in left) may contribute to increased array heterogeneity and its subsequent degeneration. The “T” in bold indicates C > T mutations.

Figure 3 outlines short (immediate) and later divergence events apparent in rDNA loci of hybrids and allopolyploids. Central early players in rDNA divergence are likely to be epigenetic mechanisms, which appear to have dual, contrasting roles. The epigenetic marks signposting active rDNA units (Fig. 3, right) with transcribed genes may contribute to array homogeneity by facilitating recombination and gene conversion. Active decondensed chromatin may also be vulnerable to DNA breaks leading to locus loss and rearrangements. In contrast, repressive epigenetic marks, such as methylcytosine (5 mC) and histone H3K9 methylation (Fig. 3, left) may not only stabilize rDNA silencing (NOR inactivity, termed nucleolar dominance in allopolyploids, reviewed in Borowska-Zuchowska et al. (2023) but may also inhibit recombination (Melamed-Bessudo and Levy 2012; Underwood et al. 2018) and sequence homogenization. Over time, 5 mC, either passively or actively, deaminates and converts into thymine (T) in what is thought to be a random process. A consequence is that recombination is further inhibited because of insufficient similarity between the units, manifesting in an incomplete concerted evolution phenotype.

### Frequency of meiotic cycles and asexual reproduction

Organisms with asexual (apomictic) reproduction might be expected to show low frequencies of concerted evolution due to a reduced rate of meiotic crossovers (Pringle et al. 2000). In support of this hypothesis, some apomictic species display increased diversity of rDNA units compared to their sexual counterparts (Fehrer et al. 2009; Machackova et al. 2022), although in other systems, differences are not so pronounced (Zaveska Drabkova et al. 2009). Elsewhere, in parthenogenetic lizards (Hillis et al. 1991) and some plant species with prevalent vegetative propagation (e.g., strawberry, onion, iris) (Fredotovic et al. 2014; Hizume et al. 2002; Lim et al. 2007; Liu and Davis 2011) or impaired meiosis (e.g., dogroses, Herklotz et al. 2018) there are normal (high) levels of rDNA homogeneity. It should be mentioned that asexual and sexual modes of reproduction may occasionally be switched in plants and ‘a little bit of sex may help to avoid genomic decay and extinction of apomictic populations’ (Hojsgaard and Hörandl 2015). Thus, rDNA diversity in apomictic species can be explained by inherited variation from past hybridization events rather than having accumulated with apomixis (discussed further below). Nevertheless, in the crustacean genus *Daphnia*, different spectra of rDNA variants can arise within 90 generations of apomictic reproduction (McTaggart et al. 2007). These observations indicate that rDNA may also be a target of some form of somatic recombination. It has been hypothesized that the nucleolus serves as a niche for this process due to intensive transcription of 45S rDNA leading to double-strand breaks (Kovarik et al. 2008). The subsequent repair process may both increase or decrease array homogeneity (Sims et al. 2021).

### The effects of life span, genetic drift and genome size

Life span may also play a role in homogenization frequency, since herbaceous plant lineages have ITS substitution rates almost twice as high as woody plants (Kay et al. 2006), which are much longer lived. There is also evidence that long-lived species such as gymnosperms display a high diversity of repeats in their genomes (Nystedt et al. 2013). Indeed, amongst gymnosperms, the cycads (genus *Cycas*) show extraordinary intragenomic rDNA heterogeneity, high rDNA copy numbers and high pseudogene content (Wang et al. 2016). It has been speculated that many gymnosperms have expanded genomes (>18 Gb/1 C on average) because of failing or reduced recombination processes that would otherwise remove non-functional DNA, especially retroelements (Leitch and Leitch 2012). Such a phenomenon might also maintain non-functional rDNA copies. However, some short-lived animals such as the grasshopper *Podisma pedestris* also show a high diversity of rDNA repeats in its large (18 Gb/1 C) genome (Keller et al. 2006). Species in *Cycas* and to a lesser extent *Podisma pedestris*, occur in relatively isolated populations today (both perhaps from much larger ancestral populations) where the effects of genetic drift are likely to be significant. These observations argue that long life spans, small population sizes, large genome sizes and infrequent meiotic cycles are associated with incomplete homogenization.

### Developmental factors and premeiotic recombination

Although generally rDNA units show faithful Mendelian inheritance, newly amplified variants of IGS not present in parental lineages have been reported among siblings in animals (Cluster et al. 1987; Reeder et al. 1976) and in lineages of synthetic allopolyploid plants (Lin et al. 1985; Skalicka et al. 2003). While these case examples remain unexplained, studies in *Xenopus laevis* (African clawed frog) point to developmental effects. In this organism, primordial germ cells amplify huge amounts of extrachromosomal rDNA through recombination which has cell-to-cell sequence variation, especially in the IGS (Kalt and Gall 1974; Bird 1978). Although extrachromosomal rDNA copies are lost during development, some of these molecules can potentially recombine with chromosomal rDNA arrays at the premeiotic stage, giving rise to new variants (Haig 2021). Concerted evolution of these variants can then increase rDNA homogeneity within the cell and increase heterogeneity among them. Variable rDNA genotypes arising from such a process can then be subjected to cellular selection in development. Selection for preadult developmental variation in intergenic spacers of X chromosome-linked rDNA loci have also been proposed in *Drosophila* (Cluster et al. 1987) and certain rDNA unit length variants have been correlated with development rates. In this context, Haig (2021) proposed that non-coding IGS sequences are subject to positive intranuclear selection for persistence and spread through arrays. It will be interesting to investigate plants, which lack true germline and determine if somatic mutations are transmitted to the next generation.

### The contribution of environmental factors to rDNA variation

There is some evidence that environmental factors may also contribute to the occurrence of rDNA variation, or perhaps rDNA variation may increase the environmental adaptability of certain organisms. This is because rDNA variation may influence rDNA transcription, which may in turn affect translation of protein coding genes and cell physiology (Kurylo et al. 2018). For instance, high level of intragenomic variation of rDNA sequences was found and postulated to be associated with adaptability to severe environments in extremotolerant and extremophilic microorganisms (Lopez-Lopez et al. 2007). Relationships between IGS variants and environmental factors such as drought, rainfall, soil composition and different habitats have also been documented in several plant systems (Cluster and Allard 1995; Govindaraju and Cullis 1992; Saghai-Maroof et al. 1984; Sharma et al. 2004) and reviewed in Nieto Feliner and Rosselló (2012). However, it remains to be determined if variation in non-coding regions of rDNA units modifies the transcriptional efficiency of units and if these have adaptive significance.

## Genetic and epigenetic barriers interfering with rDNA homogenization processes

**Fig. 4:**
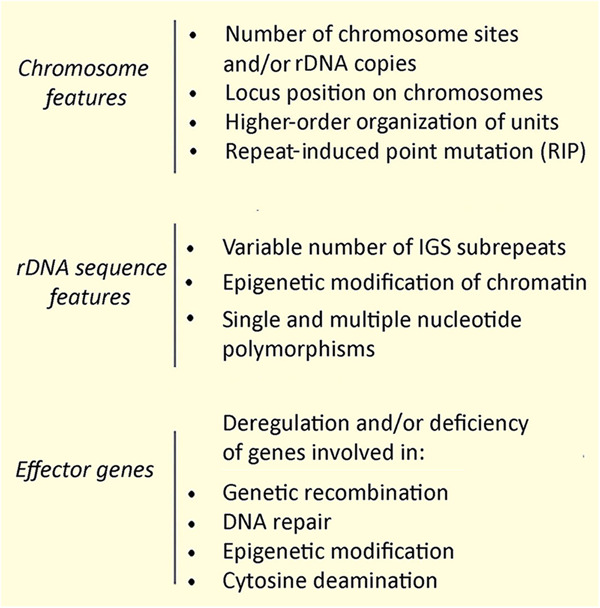
Molecular factors influencing rDNA homogeneity. They operate at different levels, through different mechanisms and vary between the organisms.

As discussed in the previous section, inhibition of recombination might lead to rDNA variation/polymorphism. Alternatively, the same outcome can be expected if the mutation rate overcomes the frequency of recombination. Indeed, individual units in tandem arrays are vulnerable to degeneration and loss of functionality, and can be blind to selection until they accumulate to such an extent that the fitness of the organism is impaired. We argue that concerted evolution functions as a correction mechanism, secondary to DNA repair, that has evolved to better control the fidelity of multigenic, tandemly repeated, families. This may be particularly significant in multicellular eukaryotic organisms whose genomes are overcrowded with repeats. It is likely that recombination would be most frequent in a homogeneous array than in an array that contains a diversity of repeats, since recombination is dependent on sequence identity. In other words, it can be hypothesized that the excision of deleterious variant(s) by recombination is more likely if they occur in a homogeneous array. This hypothesis is supported by several observations in plants, where species bearing a diversity of repeats in their genomes tend also to have tremendously expanded genome sizes (Novak et al. 2020; Nystedt et al. 2013). Based on this theoretical supposition, we suggest the following factors as potential barriers hindering homogenization and giving rise to subsequent incomplete concerted evolution (Fig. 4):

### Chromosome barriers

The number of rDNA loci could impact rDNA homogenization, since there is evidence that interlocus homogenization is less frequent than intralocus homogenization. Consequently, species with fewer rDNA loci are more likely to display complete homogenization of units than those with more rDNA loci. Indeed, the relationship between intragenomic diversity and locus number has been demonstrated in a number of plant species including *Asclepias* (Weitemier et al. 2015), *Arabidopsis* (Copenhaver and Pikaard 1996; Riddle and Richards 2005), *Nicotiana* (Matyasek et al. 2012) and *Ephedra* (Wang et al. 2016). Nevertheless, fungi bearing relatively few rDNA copies in their genomes show considerable heterogeneity of rDNA (Supplementary Table 1), and incomplete homogenization may be driven by other processes in these species (discussed further below).

Homogenization of rDNA is also likely to be influenced by the chromosome location of rDNA loci (Brownell et al. 1983). In cotton and tobacco allotetraploids, terminally located 45S rDNA loci were homogenized to near completion by interlocus gene conversion (Lim et al. 2000; Volkov et al. 1999; Wendel et al. 1995). Of note, gene conversion seems to be the only mechanism reducing rDNA diversity without changing locus numbers. Either terminal positions could be (or are) more favorable to recombination, or other positions could be (or are) unfavorable to recombination, or both. Certainly, recombination rates are not equal across the genome. However, active sites of recombination differ between species. Members of the *Mus* genus (mouse) bear high number of 45S loci with homogenized rDNA that are almost exclusively at pericentromeric position of telocentric chromosomes (Cazaux et al. 2011). Also, in Northern pike (fish) the highly amplified and homogenized 5S rDNA occur at pericentromeric positions of most telocentric/acrocentric chromosomes (Symonová et al. 2017b). Potentially the tendency towards homogenization could be determined by the physical proximity of rDNA clusters to telomeres or centromeres. The position of the individual rDNA unit within the rDNA array may also influence their likelihood to be homogenized. The edge-located copies in a single rDNA cluster apparently do not undergo homogenization or undergo it at a lower frequency, resulting in their pseudogenization in humans (Robicheau et al. 2017) and wheat (Tulpova et al. 2022). Horizontal transfers of DNA may also introduce rDNA variation. In grasses, an rDNA horizontal transfer resulted in the fast erosion of an rDNA array and its colonization by transposable elements (Mahelka et al. 2017). Conversely, another recently described rDNA horizontal transfer from the eudicot *Potentilla* to the monocot *Erythronium dens-canis* generated an intact and even partially expressed rDNA array from *Potentilla* (Bartha et al. 2022).

Theoretical models predict that the time needed for array homogenization increases roughly linearly with the initial size of the array (Smith 1974). The number of rDNA copies within eukaryotic genomes is variable but is positively correlated with genome size (Prokopowich et al. 2003). Recombination, including unequal crossing-over, can result in one recombinant chromosome having more rDNA copies and the other fewer copies. Chromosomes that have too few rDNA units might be selected against, perhaps as a result of accumulated deleterious mutations or insufficient copy numbers of functional units. Indeed, in allohexaploid wheat bearing multiple rDNA loci, the very small A-genome loci are more heterogeneous compared to larger B and D genome loci (Tulpova et al. 2022). However, too large an rDNA array may be prone to deletion mediated by intralocus recombination between distal units. Perhaps, there might be an optimal size for the array functionality, given the frequency of and position of recombination in the species.

Processes related to homology searches in DNA repair/recombination also need to be considered. In the filamentous fungus *Neurosporra crassa*, 5S rDNA unit copies can be targeted by Repeat Induced Point mutations (RIP) in the premeiotic phase of the life cycle (Selker and Stevens 1985). Loci exposed to RIP exhibit high rates of G → A and C → T transitions, elevating sequence divergence. The mechanism of repeat recognition for RIP involves direct interactions between homologous double-stranded DNA (dsDNA) segments in somatic cells (Gladyshev and Kleckner 2017). It should be noted that, in contrast to animals and plants, 5S rDNA does not form typical tandems in *N. crassa* and units are dispersed across multiple chromosomes. Nevertheless, RIP may potentially account for an unusually high level (18–83%) of 5S rDNA pseudogenes in these filamentous fungi (Rooney and Ward 2005), giving rise to deleterious mutations that are reversed by new copies that appear, consistent with the birth-and-death model of multigene family evolution (Nei and Rooney 2005). Whether RIP (or analogous mechanisms) accounts for rDNA pseudogenization in other multicellular eukaryotes remains to be determined.

Collectively, physical barriers including the position and site numbers of rDNAs on the chromosomes, chromosome rearrangements, intrinsic structure of arrays and the number of rDNA copies within one locus, are more likely functioning together rather than independently, and all are likely to influence recombination and homogenization of rDNAs.

#### rDNA structural barriers

Natural sequence variation is found in each of the rDNA unit subregions in most species (Fig. 1), although it is typically several-fold higher in IGS than in the rest of the unit (Ambrose and Crease 2011; Draisma et al. 2012; Krawczyk et al. 2017; Lunerova et al. 2017). IGS regions can be very large (up to tens of kb in some species) and provide a natural niche for alien sequence insertions, e.g. tandem repeat (sub-repeat) and even functional 5S rRNA genes (Drouin et al. 1992; Galián et al. 2012). The GC-rich minisatellites residing in many species’ IGS can be particularly polymorphic, indeed in *Cucurbita moschata* (pumpkin) IGS displays both high levels of intra- and inter-array heterogeneity (Matyasek et al. 2019). Similarly, most IGS polymorphisms in human rDNA are located at CT and TG repeated sequences (Fan et al. 2022). Indeed, much intra-genomic variation in rDNA is primarily driven by structural elements residing in the IGS, especially involving short tandem repeats.

Epigenetic modifications of chromatin, such as DNA methylation and histone modifications might occur in a step-wise manner and influence for example DNA condensation, chromatin structure, ultimately affecting recombination and homogenization processes (Potapova and Gerton 2019). The differential condensation of active vs inactive rDNA chromatin, which is driven by epigenetic status of the rDNA units, will also impact rates of recombination and hence frequency of rDNA homogenization.

#### Effector gene barriers

A large number of genes control genetic recombination, transcription, epigenetic modification, and DNA repair. The involvement of these genes should be considered to better understand mechanisms of incomplete concerted evolution of rDNAs.

Recombination and transcription-relevant genes are critical for our understanding of concerted evolution given their role in unequal crossovers and/or gene conversion and in the transcriptional activity of NORs (Cockrell and Gerton 2022). In budding yeast, it is well known that mutations in the *SIR2* (encoding histone deacetylase) and *FOB1* (a replication fork blocking factor) genes respectively increase and decrease recombination within rDNA repeats (Gottlieb and Esposito 1989; Kobayashi and Horiuchi 1996). Both genes exhibit multiple functions in a cell and particularly *SIR2* has been relatively well described. Briefly, *SIR2* encodes an NAD + -dependent histone deacetylase that catalyzes and accelerates the de-acetylation of histones H3 and H4 (Blander and Guarente 2004). Hypoacetylation of histones, limiting the accessibility of chromatin, is a heterochromatin hallmark in a wide range of organisms, from yeast to humans. With hypoacetylation of histones, rDNA transcription is silenced and the sister chromatid recombination on rDNA sites is inhibited (Smith and Boeke 1997). This may prolong the persistence of silenced parental rDNAs after the interspecific hybridization (Kovarik et al. 2008). Finally, a relationship between DNA damage and rDNA instability is evidenced by experiments in *Drosophila,* where experimentally induced DNA breaks by I-CreI endonuclease altered the rDNA copy number (Paredes and Maggert 2009).

Cytosine deamination processes are an abundant source of genetic variability in eukaryotic cells (Duncan and Miller 1980). In particular, methylated cytosines residues are mutation hot spots since deamination of 5mC leads directly to T (C → T substitution) while deamination of C leads to C → U substitutions (Fig. 3). Thus, mutation load might be higher in densely methylated genomes than in genomes with no or low levels of methylation. Indeed, the C → T transitions are the most abundant SNPs seen in plants (Buckler et al. 1997) and grasshoppers (Keller et al. 2006) rDNA. Both plants (Meyer 2011) and grasshoppers (Robinson et al. 2011) also bear high level of methylation in their DNA.

Such unit divergence is likely to inhibit homology searches and recombination (Fig. 3, left). In animals, cellular cytosine deaminase also known as (Aid)/apolipoprotein B mRNA-editing enzyme (APOBEC3) family (Pecori et al. 2022) converts C to T (or 5 mC to T) giving rise to T/G mismatches. These mismatches can be recognized and ultimately removed by the methyl-CpG-binding domain 4 (Mbd4) glycosylase or thymine DNA glycosylase (Tdg) (Kunal et al. 2008). Hypothetically, increased cytosine deaminase activity or base excision repair (BER) defects would elevate mutation rates in rDNA leading to incomplete homogenization. In *Arabidopsis thaliana* active demethylation is performed by the activity of DNA glycosylase, mainly referred to as *DEM*/*ROS1* family glycosylases and the BER pathway (Ikeda and Kinoshita 2009; Zhu 2009). Briefly, DNA glycosylases (*DME*/*ROS1* family) involved in the BER process first recognize and then directly remove various substrates, including T/G mismatches that in most cases are generated during deamination (Baute and Depicker 2008). However, the relationship between the APOBEC3 activities, (methyl)cytosine deamination and rDNA pseudogenization remain to be established. It should also be mentioned that strategies dealing with mismatches caused by cytosine deamination may differ between plants and animals (Law and Jacobsen 2010).

Taken together, we suggest that screening of the genes above and comparing across a range of species their sequences and activities with the levels of rDNA polymorphisms, would lead to a better understanding of the processes leading to incomplete concerted evolution of rDNAs.

## Conclusions and perspectives

In the vast majority of eukaryotes, evidence of concerted evolution is observed in rDNAs arrays. However, numerous examples of rDNA sequence and copy number variations observed in a wide range of genera and in different subregions of rDNA units raise several questions, which include: can unbiased criteria discriminate between complete, incomplete, and even failing concerted evolution? To what extent (threshold) can intragenomic variation/polymorphisms in rDNA units be attributed to “incomplete” concerted evolution? How might we discriminate variants that could go to fixation (functional) from those that contribute to array heterogeneity and are non-functional? How strong is selection along the different sequence categories of rDNA units? What is the level of unit-to-unit variation within active and non-active arrays? What is the relationship between rDNA copy number and nucleotide variation?

In order to address these questions, it will be essential to improve methodical approaches used for scoring rDNA variation within and across the arrays establishing clearly defined, biologically relevant threshold values, e.g., specific number of variations, SNPs per DNA sequence length. MinION technology, generating >200 kb reads, appears to be a suitable method for long-scale analyses of rDNA arrangement and unit structure (McKinlay et al. 2021), as was applied to the latest human T2T genome assembly including rDNA assembly (Nurk et al. 2022). However, its per-base error rate is still high (5–15%) (Istace et al. 2017) preventing unambiguous base calling. Therefore, assembling the highly repetitive rDNAs at least covering several 18S-5.8S-28S-IGS units (each of these “units” has a size of c. 10–50 kb in general) with lower sequencing errors, requires with expensive and high coverage sequencing. The HiFi PacBio technology (PacBio Biosciences, USA) generating long reads seems to overcome the accuracy problem (the claimed accuracy of >99% is comparable to short reads or Sanger sequencing) and could become a method of choice for determining single nucleotide variation within rDNA arrays. In the course of manuscript revision a near complete reconstruction of *Arabidopsis thaliana* 5S rDNA clusters by employing the above-mentioned approach was reported (Rabanal et al. 2022). We believe that the phenomenon and potential mechanisms of incomplete concerted evolution of rDNA identified here may only represent the tip of the iceberg to fully understand the evolution and functional diversity of rDNA in the future.

## Supplementary information

Supplementary Table S1

Supplementary Table S2
