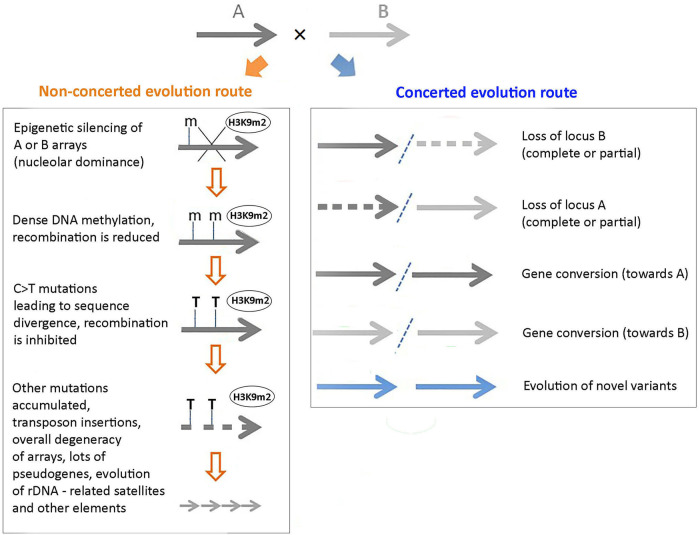# Correction: Intragenomic rDNA variation—the product of concerted evolution, mutation, or something in between?

**DOI:** 10.1038/s41437-023-00644-3

**Published:** 2023-08-02

**Authors:** Wencai Wang, Xianzhi Zhang, Sònia Garcia, Andrew R. Leitch, Aleš Kovařík

**Affiliations:** 1 Science and Technology Innovation Center, Guangzhou University of Chinese Medicine, Guangzhou, 510405 China; 2 Department of Horticulture, College of Horticulture and Landscape Architecture, Zhongkai University of Agriculture and Engineering, Guangzhou, 510225 China; 3 Institut Botànic de Barcelona, IBB (CSIC - Ajuntament de Barcelona), Barcelona, Spain; 4 School of Biological and Behavioral Sciences, Queen Mary University of London, London, E1 4NS UK; 5 Institute of Biophysics, Academy of Sciences of the Czech Republic, Brno, CZ–61200 Czech Republic

**Keywords:** Evolutionary genetics, Evolutionary biology, Plant evolution

Correction to: *Heredity* 10.1038/s41437-023-00634-5, published online 04 July 2023

In this article, Figure 3 has been incorrectly given. The figure should read as follows. The original article has been corrected.