# Supplementary material for: Intragenomic rDNA variation - the product of concerted evolution, mutation, or something in between?
Source: Heredity (Edinb). 2023 Jul 4;131(3):179–88. doi: 10.1038/s41437-023-00634-5 (PMC10462631; doi:10.1038/s41437-023-00634-5)
Supplement: Supplementary file 1 — Supplementary Table S1 [file 41437_2023_634_MOESM1_ESM.pdf]

Supplementary Table S1. List of genera/species showing intragenomic variation in the rDNA units

No.	Phylum	Species/Genus/Family	rDNA type/sub region[1]	Method used for estimation of rDNA variation	Locus number (45S/5S) per diploid genome[2]	45S and 5S copy number per haploid genome[3]	References
1	animal	*Nassellari and Spumellariaa*	45S	Sanger sequencing, Illumina sequencing and Oxford Nanopore Technologies (MinION).	-/-	-/-	Sandin MM, Romac S, Not F. (2022) Intra-genomic rRNA gene variability of Nassellaria and Spumellaria (Rhizaria, Radiolaria) assessed by Sanger, MinION and Illumina sequencing. Environ Microbiol. doi: 10.1111/1462-2920.16081.
2	animal	*Homo sapiens*	45S	Whole genome sequencing projects	10/2	~217/~250[4]	Fan WJ, Eklund E, Sherman RM, et al. (2022) Widespread genetic heterogeneity of human ribosomal RNA genes, RNA, 28(4):478-492.
3	animal	*Anopheles stephensi*	ITS2	Cloning, Sanger sequencing, qPCR)	2/-	-/-	Mishra S, Sharma G, Das MK, et al. (2021) Intragenomic sequence variations in the second internal transcribed spacer (ITS2) ribosomal DNA of the malaria vector Anopheles stephensi. bioRxiv preprint doi: https://doi.org/10.1101/2021.04.05.437320
4	animal	*Lubomirskia baikalensis*	ITS	Sanger sequencing	2/-	-/-	Itskovich V, (2020) Intragenomic variation of rDNA internal transcribed spacers in the endemic Baikal sponge Lubomirskia baikalensis (Pallas, 1776) (Spongillida, Lubomirskiidae): Implications for Porifera barcoding. Journal of Great Lakes Research, 46(1):62-66

5	animal	*Paraplagusia blochii*	ITS	PCR, cloning, Sanger sequencing	2/-	-/-	Gong L, Kong XY, Luo HR, et al. (2020) Intra-genomic variability and pseudogenes in ribosomal ITS regions of Paraplagusia blochii (Pleuronectiformes: Cynoglossidae). Animal Biology, 70(2):145-158
6	animal	*Diolcogaster*	ITS2	Next generation sequencing	2-12/-	-/-	Fagan-Jeffries EP, Cooper SJB, Bradford TM, Austin AD (2019) Intragenomic internal transcribed spacer 2 variation in a genus of parasitoid wasps (Hymenoptera: Braconidae): implications for accurate species delimitation and phylogenetic analysis. Insect Molecular Biology, 28(4):485-498
7	animal	*Pleuronichthys cornutus*	18S	PCR, cloning, Sanger sequencing	-/-	-/-	Yang M, Kong XY, Shi W, et al. (2018) Remarkable sequence polymorphisms in 18S rDNA of Pleuronichthys cornutus (Pleuronectiformes Pleuronectidae). Gene 677:251-258
8	animal	*Clonorchis sinensis*	ITS	PCR, Sanger sequencing	-/-	-/-	Yulia V. Tatonova, Galina N. et al. (2017) Inter-individual and intragenomic variations in the ITS region of Clonorchis sinensis (Trematoda: Opisthorchiidae) from Russia and Vietnam.Infection, Genetics and Evolution, 55:350-357
9	animal	*Scirtothrips dorsalis,Thrips palmi, Frankliniella occidentalis*	ITS2	PCR, Sanger sequencing	-/-	-/-	Kumar V, Dickey AM, Seal DR, et al. (2017) Unexpected high intragenomic variation in two of three major pest thrips species does not affect ribosomal internal transcribed spacer 2 (ITS2) utility for thrips identification. International Journal of Molecualr Sciences, 18:2100, doi:10.3390/ijms18102100

10	animal	*Ripella platypodia, R.decalvata.R.tribonemae*	18S	PCR, cloning, Sanger sequencing	-/-	-/-	Kudryavtsev A and Gladkikh A. (2017) Two new species of Ripella (Amoebozoa, Vannellida) and unusual intragenomic variability in the SSU rRNA gene of this genus. Eur J Protisto,61(Pt A):92-106.
11	animal	*Cephalenchus*	ITS, 28S	PCR, cloning, Sanger sequencing	-/-	-/-	Pereira TJ and Baldwin JG (2016) Contrasting evolutionary patterns of 28S and ITS rRNA [illegible]
12	animal	*Cynoglossus zanzibarensis*	ITS2	PCR, cloning, Sanger sequencing	-/-	-/-	Gong L, Shi W, Yang M, et al. (2016)Non-concerted evolution in ribosomal ITS2 sequence in Cynoglossus zanzibarensis (Pleuronectiformes: Cynoglossidae). Biochemical Systematics and Ecology 66:181-187.
13	animal	*Agrodiaetus*	ITS2	PCR, cloning, Sanger sequencing	-/-	-/-	Shapoval NA and Lukhtanov VA.(2015) Intragenomic variations of multicopy ITS2 marker in Agrodiaetus blue butterflies (Lepidoptera, Lycaenidae). Comparative Cytogenetics. 9(4):483-97.
14	animal	*Pocillo pora damicornis*	ITS2	End-point PCR, Denaturing Gradient Gel Electrophoresis(DGGE), qPCR, Sanger sequencing,	-/-	-/-	Wilkinson SP, Fisher PL, van Oppen MJ, et al. (2015) Intra-genomic variation in symbiotic dinoflagellates: recent divergence or recombination between lineages? BMC Evol Biol.15:46
15	animal	*Eyprepocnemis plorans*	ITS2	Tagged PCR 454 amplicon sequencing	2-4/-	-/-	Ruiz-Estévez M, Ruiz-Ruano FJ, Cabrero J, et al.(2015) Non-random expression of ribosomal DNA units in a grasshopper showing high intragenomic variation for the ITS2 region. Insect Molecular Biology. 24(3):319-30.

16	animal	*Foraminifera*	18S	PCR, cloning, Sanger sequencing	-/-	-/-	Weber AA, Pawlowski J.(2014) Wide occurrence of SSU rDNA intragenomic polymorphism in foraminifera and its implications for molecular species identification. Protist. 2014 Sep;165(5):645-61
17	animal	*Potamopyrgus antipodarum*	ITS-18S-2	PCR, cloning, Sanger sequencing	-/-	-/-	Marshal SH, Rusty JR, (2013) Intragenomic sequence variation at the ITS1–ITS2 region and at the 18S and 28S nuclear ribosomal DNA genes of the New Zealand mud snail, Potamopyrgus antipodarum (Hydrobiidae: Mollusca), Journal of Molluscan Studies, 79(3) : 205–217
18	animal	*Caenorhabditis elegans, C. briggsae, C. japonica, C.remanei*	18S-ITS1-	Whole genome sequencing projects	2/-	~55/-	Bik HM, Fournier D, Sung W, et al.(2013) Intra-genomic variation in the ribosomal repeats of nematodes. PLoS One. 8(10):e78230.
19	animal	*Ephydatia fluviatilis*	ITS	PCR, cloning, Sanger sequencing	-/-	-/-	Karlep L, Reintamm T, Kelve M.(2013) Intragenomic profiling using multicopy genes: the rDNA internal transcribed spacer sequences of the freshwater sponge Ephydatia fluviatilis. PLoS One.8(6):e66601
20	animal	*Elphidium macellum*	18S	PCR, cloning, Sanger sequencing	-/-	-/-	Pillet L, Fontaine D, Pawlowski J.(2012) Intra-genomic ribosomal RNA polymorphism and morphological variation in Elphidium macellum suggests inter-specific hybridization in foraminifera. PLoS One.7(2):e32373

21	animal	*Khawia*	ITS2	PCR, cloning, Sanger sequencing	-/-	-/-	Králová-Hromadová I, Bazsalovicsová E, Oros M, et al. (2012). Sequence structure and intragenomic variability of ribosomal ITS2 in monozoic tapeworms of the genus Khawia (Cestoda: Caryophyllidea), parasites of cyprinid fish. Parasitol Res. 111(4):1621-7
22	animal	*Culex and Lutzia*	ITS2	PCR, cloning, Sanger sequencing	2/-	-/-	Vesgueiro FT, Demari-Silva B, Malafronte Rdos S, et al. (2011) Intragenomic variation in the second internal transcribed spacer of the ribosomal DNA of species of the genera Culex and Lutzia (Diptera: Culicidae). Mem Inst Oswaldo Cruz.106(1):1-8
23	animal	*Atractolytocestus huronensis*	ITS2	PCR, cloning, Sanger sequencing	3/-*	-/-	Bazsalovicsová E, Králová-Hromadová I, Stefka J, et al.(2011) Population study of Atractolytocestus huronensis (Cestoda: Caryophyllidea), an invasive parasite of common carp introduced to Europe: mitochondrial cox1 haplotypes and intragenomic ribosomal ITS2 variants. Parasitol Res.109(1):125-31
24	animal	*Cyclospora cayetanensis*	ITS1	PCR, cloning, Sanger sequencing	-/-	11/-[5]	Riner DK, Nichols T, Lucas SY, et al. (2010) Intragenomic sequence variation of the ITS-1 region within a single flow-cytometry-counted Cyclospora cayetanensis oocysts. J Parasitol. 96(5):914-919
25	animal	*Anopheles longirostris*	ITS2	RFLP,PCR, cloning, Sanger sequencing	2/-	-/-	Alquezar DE, Hemmerter S, Cooper RD, et al. (2010) Incomplete concerted evolution and reproductive isolation at the rDNA locus uncovers nine cryptic species within Anopheles longirostris from Papua New Guinea. BMC Evolutionary Biology 10(1):392.

26	animal	*Kareius bicoloratus*	18S-ITS1	PCR, cloning, Sanger sequencing	-/-	-/-	Xu J, Zhang Q, Xu X, et al.(2009) Intragenomic variability and pseudogenes of ribosomal DNA in stone flounder Kareius bicoloratus. Mol Phylogenet Evol.52(1):157-66
27	animal	*Cumberlandia monodonta*	ITS1	PCR, cloning, Sanger sequencing	-/-	-/-	Elderkin CL. (2009) Intragenomic variation in the rDNA internal transcribed spacer (ITS1) in the freshwater mussel Cumberlandia monodonta (Say, 1828). Journal of Molluscan Studies, 75:419-421
28	animal	*Caribbean seafan octocorals Gorgonia and Pseudopterogorgia*	ITS2	DGGE, PCR, Sanger sequencing, RNA secondary structure prediction	-/-	-/-	Sanchez JA and Dorado D, (2008) Intragenomic ITS2 variation in Caribbean seafans, Proceedings of the 11th International Coral Reef Symposium, Florida, 7-11, July 2008
29	animal	*Pacifigorgia spp.* and *Leptogorgia spp.*	ITS2	DGGE PCR, Sanger sequencing, RNA secondary structure prediction	-/-	-/-	Granados Cifuentes CA, (2008) Interspecific and intragenomic variation of the internal transcribed spacer2 (ITS2, rDNA) and their consequence in the evolution of eastern pacific octocorals. Master Thesis
30	animal	*Anopheles messeae*	ITS2	PCR, cloning, Sanger sequencing	2/-	-/-	Bezzhonova OV, Goryacheva II. (2008) Intragenomic heterogeneity of rDNA internal transcribed spacer 2 in Anopheles messeae (Diptera: Culicidae). J Med Entomol. 2008 May;45(3):337-41
31	animal	*Anopheles (Nyssorhynchus) albitarsis complex*	ITS2	PCR, cloning, Sanger sequencing	2/-	-/-	Li C and Wilkerson RC (2007). Intragenomic rDNA ITS2 variation in the neotropical Anopheles (Nyssorhynchus) albitarsis complex (Diptera: Culicidae). J Hered. 98(1):51-59

32	animal	*Acropora valida*	ITS	PCR-DGGE, direct Sanger sequencing	2/2	-/-	LaJeunesse TC, Pinzón JH. (2007) Screening intragenomic rDNA for dominant variants can provide a consistent retrieval of evolutionarily persistent ITS (rDNA) sequences. Mol Phylogenet Evol. 45(1):417-422
33	animal	*Halichondrida*	ITS	PCR, cloning, Sanger sequencing	-/-	-/-	Alvarez B, Krishnan M, Gibb K, (2007) Analysis of intragenomic variation of the rDNA internal transcribed spacers (ITS) in Halichondrida (Porifera: Demospongiae). J. Mar. Biol. Ass. U.K. 87:1599-1605
34	animal	*Porites, Siderastrea*	ITS	PCR, cloning, Sanger sequencing	-/-	-/-	Forsman ZH, Hunter CL, Fox GE, et al. (2006) Is the ITS region the solution to the “species problem” in corals? Intragenomic variation and alignment permutation in Porites, Siderastrea and outgroup taxa. Proceedings of the 10th international coral reef symposium.1: 14-23.
35	animal	*Podisma pedestris*	18S-ITS1	PCR, RT-PCR, Southern blot, Sanger sequencing	3-13/-	-/-	Keller I, Chintauan-Marquier IC, Veltsos P. et al. (2006) Ribosomal DNA in the grasshopper Podisma pedestris: escape from concerted evolution. Genetics 174(2):863.
36	animal	*Anopheles aquasalis*	ITS	PCR, cloning, Sanger sequencing	2/-	-/-	Fairley TL, Kilpatrick CW, Conn JE.(2005) Intragenomic heterogeneity of internal transcribed spacer rDNA in neotropical malaria vector Anopheles aquasalis (Diptera: Culicidae). J Med Entomol. 42(5):795-800
37	animal	*Lytechinus variegatus*	IGS (5S)	EM denaturation mapping	/	-/-	Mishra NK.(2005) Intragenomic variation in ribosomal RNA gene of the sea urchin Lytechinus variegatus. Mol Biol Rep. 32(1):61-65

38	animal	*Phylum Porifera*	ITS	PCR, cloning, Sanger sequencing	/	-/-	Wrheide G , Nichols SA , Goldberg J.(2004) Intragenomic variation of the rDNA internal transcribed spacers in sponges (Phylum Porifera): implications for phylogenetic studies. Molecular Phylogenetics & Evolution, 33(3):816-830
39	animal	*Hymeniacidon heliophila*	ITS1, ITS2	PCR, Polymerase chain reaction-based single-strand conformation polymorphism method(PCR-SSCP)	-/-	-/-	Lôbo-Hajdu G, Guimarase ACR, Salgado A, et al. (2004) IntragenomicIntra-and interspecific variation in the rDNA its of Porifera revealed by PCR-singlestrand conformation polymorphism (PCR-SSCP). Boll. Mus. Ist. Biol. Univ. Genova., 68:413-423
40	animal	*Homo sapines*	IGS (45S)	PCR, cloning, Sanger sequencing	10/2	-/-	Shibalev DV, Voronov AS, Firsov SIu, et al. (2004) Detection of intragenomic polymorphism in the LR2 region of human intergenic ribosomal spacer]. Mol Biol (Mosk). 38(6):980-984
41	animal	*Sidalcea*	ITS, IGS (	PCR, cloning, Sanger sequencing	-/-	-/-	Andreasen K, and Baldwin BG (2003) Nuclear ribosomal DNA sequence polymorphism and hybridization in checker mallows (Sidalcea, Malvaceae). Molecular Phylogenetics and Evolution 29(3):563-581.
42	animal	*Acropora*	ITS1-5.8S	RT-PCR, Sanger sequencing	-/-	-/-	Márquez LM, Miller DJ, MacKenzie JB, et al. (2003) Pseudogenes contribute to the extreme diversity of nuclear ribosomal DNA in the hard coral Acropora. Molecular Biology and Evolution 7:1077-1086.
43	animal	*Pediculus humanus*	ITS2	PCR, cloning, Direct cycle-sequencing	-/-	-/-	Leo NP, Barker SC. (2002) Intragenomic variation in ITS2 rDNA in the louse of humans, Pediculus humanus: ITS2 is not a suitable marker for population studies in this species. Insect Mol Biol.11(6):651-657

44	animal	*Anopheles sinensis*	IGS (45S)	PCR, cloning, Sanger sequencing	2/-	~2000/-	Whang IJ, Jung J, Park JK, et al. (2002) Intragenomic length variation of the ribosomal DNA intergenic spacer in a malaria vector, Anopheles sinensis. Mol Cells. 14(1):158-162
45	animal	*Decapoda*	ITS1, ITS2	PCR, cloning, Sanger sequencing	2-10/-	-/-	Harris DJ, Crandall KA. (2000) Intragenomic variation within ITS1 and ITS2 of freshwater crayfishes (Decapoda: Cambaridae): implications for phylogenetic and microsatellite studies. Mol Biol Evol. 7(2):284-291
46	animal	*Anopheles nuneztovari*	ITS2	PCR, cloning, Sanger sequencing	2/-	-/-	Onyabe DY and Conn JE (2001) Intragenomic heterogeneity of a ribosomal DNA spacer (ITS2) varies regionally in the neotropical malaria vector Anopheles nuneztovari (Diptera: Culicidae). Insect Molecular Biology, 8(4):435-442
47	animal	*Myzus persicae*	ITS	PCR, cloning, Sanger sequencing	2/-	-/-	Fenton B, Malloch G, Germa F.(1998) A study of variation in rDNA ITS regions shows that two haplotypes coexist within a single aphid genome. Genome 41(3):337-345.
48	animal	*Dugesia(Schmidtea) mediterranea*	18S	PCR, direct Sanger sequencing, Southern blot, Northern blot analyses	-/-	-/-	Carranza S. Giribet G. Ribera C. et al (1996) Evidence that two types of 18S rDNA coexist in the genome of Dugesia (Schmidtea) mediterranea (Platyhelminthes, Turbellaria, Tricladida). Molecular Biology and Evolution 6:824.
49	prokaryot	*Cacao seed endophytic bacteria*	16S	PCR, Sanger sequencing and restriction analyses	-/-	<16/-	da Silva CB, dos Santos HRM, Marbach PAS, et al. (2019). First-tier detection of intragenomic 16S rRNA gene variation in culturable endophytic bacteria from cacao seeds. PeerJ 7:e7452 http://doi.org/10.7717/peerj.7452

50	prokaryot	*Haloarcula marismortui*	16S	qPCR and structural analyses	-/-	3/3	López-López A, Benlloch S, Bonfá M, et al. (2007). Intragenomic 16S rDNA divergence in Haloarcula marismortui is an adaptation to different temperatures. J Mol Evol.65(6):687-696.
51	prokaryot	*several species*	16S	Whole genome sequencing projects	-/-	-/-	Sun DL, Jiang X, Wu QL, et al. (2013). Intragenomic heterogeneity of 16S rRNA genes causes overestimation of prokaryotic diversity. Appl Environ Microbiol. 79(19):5962-5969
52	prokaryot	*several species*	ITS	Whole genome sequencing projects	-/-	3-16/-	Stewart FJ, Cavanaugh CM. (2007) Intragenomic variation and evolution of the internal transcribed spacer of the rRNA operon in bacteria. J Mol Evol. 65(1):44-67
53	fungi	*Saccharomyces cerevisiae*	35S	Whole genome sequencing projects	2/2	54-511/54-511	Sultanov D, and Hochwagen A, (2022) Deep selection shapes the intragenomic diversity of rRNA genes. BioRxiv preprint doi: https://doi.org/10.1101/2022.03.28.486124
54	fungi	*Cordyceps*	ITS	Amplicon sequencing via PacBio SEQUEL I	-/-	-/-	Paloi S, Mhuantong W, Luangsa-ard JJ et al., (2021) Using high-throughput amplicon sequencing to evaluate intragenomic variation and accuracy in species identification of Cordyceps species. Journal of Fungi, 7, 767. https://doi.org/10.3390/jof7090767
55	fungi	*Hypoxylaceae*	ITS	Illumina MiSeq sequencing, Oxford Nanopore MinION sequencing, PacBio sequencing	2/-	-/-	Stadler M, Lambert C, Wibberg D et al. (2020) Intragenomic polymorphisms in the ITS region of high-quality genomes of the Hypoxylaceae (Xylariales, Ascomycota). Mycol Progress 19, 235–245. https://doi.org/10.1007/s11557-019-01552-9

56	fungi	*Boletus edulis*	ITS	PCR, Sanger sequencing,nanopore sequencing, bioinformatics analyses	-/-	~80/-	Tremble K, Suz LM, Dentinger BTM. (2020) Lost in translation: Population genomics and long-read sequencing reveals relaxation of concerted evolution of the ribosomal DNA cistron. Mol Phylogenet Evol. 148:106804
57	fungi	*Tuber aestivum*	ITS	PCR, direct Sanger sequencing	-/-	-/-	Riccioni C, Rubini A, Turkoglu A, et al. (2019) Ribosomal DNA polymorphisms reveal genetic structure and a phylogeographic pattern in the Burgundy truffle Tuber aestivum Vittad. Mycologia 11(1):26-39
58	fungi	*Amanita cf. lavendula taxon 1.*	ITS	Sanger sequencing, cloning	-/-	~60/-	Hughes KW, Tulloss RH, Petersen R (2018) Intragenomic nuclear RNA variation in a cryptic Amanita taxon. Mycologia, 110(1): 93-103
59	fungi	*Rhizophagus irregularis*	45S	PacBio sequencing, Illumina sequencing, Bioinformatics	-/-	10-11/-	Maeda T, Kobayashi Y, Kameoka H, et al. (2018) Evidence of non-tandemly repeated rDNAs and their intragenomic heterogeneity in Rhizophagus irregularis. Communications Biology, 1:87
60	fungi	*Cordyceps militaris CM01;Epichloë amarillans E57; E. brachyelytri E4804 ; E. typhina;Colletotrichum graminicola M1.001;Leptosphaeria maculans JN3;Neurospora crassa*	18S-ITS-2	Whole genome sequencing	-/-	1-69/-	Li Y, Yang RH, Jiang L, et al. (2017) rRNA pseudogenes in filamentous ascomycetes as revealed by genome data. G3 (Bethesda). 7(8):2695-2703.

61	fungi	*Zygosaccharomyces rouxii*	ITS1,ITS2	PCR-RFLP, cloning,Sanger sequencing	-/-	-/-	Chand Dakal T, Giudici P, Solieri L (2016) Contrasting Patterns of rDNA Homogenization within the Zygosaccharomyces rouxii Species Complex.PLoS ONE 11(8): e0160744
62	fungi	*Pichia membranifaciens*	ITS1, ITS2	PCR, cloning, Sanger sequencing	-/-	~60/-	Wu ZW, Wang QM, Liu XZ, et al.(2016) Intragenomic polymorphism and intergenomic recombination in the ribosomal RNA genes of strains belonging to a yeast species Pichia membranifaciens. Mycology.7(3):102-111
63	fungi	*Candida glabrata, Picha norvegensis, Candida tropicalis, Saccharomyces cerevisiae*	ITS1/ITS2	PCR, cloning, Sanger sequencing	-/-	50-500/-	Zhao Y, Tsang CC, Xiao M, et al. (2015) Intra-genomic internal transcribed spacer region sequence heterogeneity and molecular diagnosis in clinical microbiology. International Journal of Molecular Sciences, 16:2506-2579
64	fungi	*Ceratocystis fimbriata sensu stricto*	ITS	PCR, cloning, Sanger sequencing	-/-	-/-	Harrington TC, Kazmi MR, Al-Sadi AM, et al. (2014) Intraspecific and intragenomic variability of ITS rDNA sequences reveals taxonomic problems in Ceratocystis fimbriata sensu stricto. Mycologia.106(2):224-42
65	fungi	*Laetiporus cincinnatus,L. huroniensis, Aspergillus sp., Annulohypoxylon multiforme, Saccharomyces cerevisiae*	ITS1	454 amplicon pyrosequencing	-/-	50-500/-	Lindner DL, Carlsen T, Henrik Nilsson R, et al. (2013) Employing 454 amplicon pyrosequencing to reveal intragenomic divergence in the internal transcribed spacer rDNA region in fungi. Ecol Evol.3(6):1751-64

66	fungi	*Ophiocordyceps sinensis*	ITS	PCR, Sanger sequencing	-/-	-/-	Li Y, Jiao L, Yao YJ, (2013) Non-concerted ITS evolution in fungi, as revealed from the important medicinal fungus Ophiocordyceps sinensis. Molecular Phylogenetics and Evolution 68(2):373-379.
67	fungi	*Neonothopanus nambi*	ITS	PCR, cloning, Sanger sequencing	-/-	-/-	Vydryakova GA, Van DT , Shoukouhi P , et al.(2012) Intergenomic and intragenomic ITS sequence heterogeneity in Neonothopanus nambi (Agaricales) from Vietnam, Mycology, 3:2, 89-99
68	fungi	*Laetiporus*	ITS	PCR, cloning, Sanger sequencing	-/-	-/-	Lindner DL, Banik MT. (2011) Intragenomic variation in the ITS rDNA region obscures phylogenetic relationships and inflates estimates of operational taxonomic units in genus Laetiporus. Mycologia.103(4):731-740
69	fungi	*Geotrichum candidum*	ITS1–5.8S	PCR, cloning, Sanger sequencing	-/-	-/-	Alper I, Frenette M, Labrie S. (2011) Ribosomal DNA polymorphisms in the yeast Geotrichum candidum. Fungal Biol. 115(12):1259-1269
70	fungi	*Sinorhizobium fredii*	16S-23S	PCR-RFLP, Sanger sequencing, Southern blot hybridization	-/-	3/-	Saeki Y, Oguro H , Akagi I, et al. (2009) Intragenomic variation in the internal transcribed spacer regions between 16S–23S rRNA genes among the three copies of Sinorhizobium fredii strains, Soil Science and Plant Nutrition, 55:5, 627-633
71	fungi	*Saccharomyces cerevisiae*	45S	WGS data+ Bioinformatics	2/2	54-511/-	James SA, O'Kelly MJT, Carter DM, et al. (2009)Repetitive sequence variation and dynamics in the ribosomal DNA array of Saccharomyces cerevisiae as revealed by whole-genome resequencing. Genome Research 19(4), 626-635.

72	fungi	*Aspergillus nidulans*	18S-ITS-2	PCR, cloning, Sanger sequencing	-/-	45/-	Simon UK and Weiss M (2008). Intragenomic variation of fungal ribosomal genes is higher than previously thought. Mol Biol Evol. 25(11):2251-4
73	fungi	*Teratosphaeria microspora*	18S-ITS-2	PCR, cloning, Sanger sequencing	-/-	-/-	Simon UK and Weiss M (2008). Intragenomic variation of fungal ribosomal genes is higher than previously thought. Mol Biol Evol. 25(11):2251-4
74	fungi	*Mycosphaerella punctiformis, Phoma exigua var. exigua, Davidiella allicina*	18S-ITS-2	PCR, cloning, Sanger sequencing	-/-	-/-	Simon UK and Weiss M (2008). Intragenomic variation of fungal ribosomal genes is higher than previously thought. Mol Biol Evol. 25(11):2251-4
75	fungi	*Phoma exigua var. exigua, Davidiella allicina*	18S-ITS-2	PCR, cloning, Sanger sequencing	-/-	-/-	Simon UK and Weiss M (2008). Intragenomic variation of fungal ribosomal genes is higher than previously thought. Mol Biol Evol. 25(11):2251-4
76	fungi	*Davidiella allicina*	18S-ITS-2	PCR, cloning, Sanger sequencing	-/-	-/-	Simon UK and Weiss M (2008). Intragenomic variation of fungal ribosomal genes is higher than previously thought. Mol Biol Evol. 25(11):2251-4
77	fungi	*Trichophyton violaceum*	IGS (45S)	RFLP,DAPI staining, Southern blot, PCR, cloning, Sanger sequencing	-/-	~60/-	Chang JC, Hsu MM, Barton RC, et al. (2008) High-frequency intragenomic heterogeneity of the ribosomal DNA intergenic spacer region in Trichophyton violaceum. Eukaryot Cell.7(4):721-726
78	fungi	*Ashbya gossypii*	45S	Shotgun sequencing data, Bioinfromatics analysis	-/-	-/-	Ganley AR, Kobayashi T. (2007) Highly efficient concerted evolution in the ribosomal DNA repeats: total rDNA repeat variation revealed by whole-genome shotgun sequence data. Genome Res.17(2):184-191

79	fungi	*Saccharomyces cerevisiae*	45S	Shotgun sequencing data, Bioinfromatics analysis	2/2	54-511/-	Ganley AR, Kobayashi T. (2007) Highly efficient concerted evolution in the ribosomal DNA repeats: total rDNA repeat variation revealed by whole-genome shotgun sequence data. Genome Res.17(2):184-191
80	fungi	*Saccharomyces paradoxus*	45S	Shotgun sequencing data, Bioinfromatics analysis	-/-	-/-	Ganley AR, Kobayashi T. (2007) Highly efficient concerted evolution in the ribosomal DNA repeats: total rDNA repeat variation revealed by whole-genome shotgun sequence data. Genome Res.17(2):184-191
81	fungi	*Aspergillus nidulans*	45S	Shotgun sequencing data, Bioinfromatics analysis	-/-	45/-	Ganley AR, Kobayashi T. (2007) Highly efficient concerted evolution in the ribosomal DNA repeats: total rDNA repeat variation revealed by whole-genome shotgun sequence data. Genome Res.17(2):184-191
82	fungi	*Cryptococcus neoformans*	45S	Shotgun sequencing data, Bioinfromatics analysis	-/-	-/-	Ganley AR, Kobayashi T. (2007) Highly efficient concerted evolution in the ribosomal DNA repeats: total rDNA repeat variation revealed by whole-genome shotgun sequence data. Genome Res.17(2):184-191
83	fungi	*Xanthophyllomyces*	ITS	PCR, cloning, Sanger sequencing	-/-	-/-	Fell JW, Scorzetti G, Statzell-Tallman A, et al.(2007) Molecular diversity and intragenomic variability in the yeast genus Xanthophyllomyces: the origin of Phaffia rhodozyma? FEMS Yeast Res. 7(8):1399-408
84	fungi	*Skeletonema*	18S	PCR, cloning, Sanger sequencing	-/-	-/-	Alverson AJ and Kolnick L.(2005) Intragenomic nucleotide polymorphism among small subunit (18s) rDNA paralogs in the diatom genus skeletonema (bacillariophyta) 1.Journal of Phycology, 41(6): 1248-1257

85	fungi	*Tricophyton mentagrophytes*	ITS	RFLP-PCR, Sanger sequencing	-/-	-/-	Mochizuki T, Ishizaki H, Barton RC, et al. (2003). Restriction fragment length polymorphism analysis of ribosomal DNA intergenic regions is useful for differentiating strains of Trichophyton mentagrophytes. Journal of Clinical Microbiology 41(10):4583-4588.
86	fungi	*Streptomyces ambofaciens*	18S, 28S	PCR, cloning, Sanger sequencing	-/-	3/-	Wenner T, Roth V, Decaris B, et al. (2002). Intragenomic and intraspecific polymorphism of the 16S-23S rDNA internally transcribed sequences of Streptomyces ambofaciens. Microbiology (Reading).148(Pt 3):633-642.
87	fungi	*Schizophyllum commune*	IGS (45S)	PCR, resetrication enzymes digestigation analysis, Sanger sequencing	-/-	-/-	James TY, Moncalvo JM, Li S, et al. (2001) Polymorphism at the ribosomal DNA spacers and its relation to breeding structure of the widespread mushroom Schizophyllum commune. Genetics 157(1):149-161.
88	fungi	*Acaulospora colossica*	ITS	PCR, cloning, Sanger sequencing	-/-	-/-	Pringle et al. 2000. High levels of variation in ribosomal DNA sequences within and among spores of a natural population of the arbuscular mycorrhizal fungus Acaulospora colossica. Mycologia 92(2), 259-268.
89	fungi	*Colletotrichum lindemuthianum*	ITS1, ITS2	PCR-RFLP analysis, Sanger sequencing	-/-	-/-	Balardin RS, Smith JJ, Kelly JD (1999) Ribosomal DNA polymorphism in Colletotrichum lindemuthianum. Mycological Research 103(7):841–848.
90	fungi	*Gibberella fujikuroi complex*	ITS2	PCR, Sanger sequencing	-/-	-/-	O'Donnell K and Cigelnik E (1997) Two divergent intragenomic rDNA ITS2 types within a monophyletic lineage of the fungus Fusarium are nonorthologous. Mol Phylogenet Evol. 7(1):103-116

91	plant	*Haematococcus pluvialis*	ITS2	PCR, Illumina MiSeq sequencing	-/-	-/-	Alanagreh L, Pegg C, Harikumar A, et al. (2017) Assessing intragenomic variation of the internal transcribed spacer two: Adapting the Illumina metagenomics protocol.PLoS ONE 12(7): e0181491
92	plant	*Pistia stratiotes*	IGS (35S,	PCR, cloning, Sanger sequencing, FISH	2/2	-/-	Stepanenko A. Chen G.Hoang PTN, et al. (2022) The ribosomal DNA loci of the ancient monocot Pistia stratiotes L. (Araceae) contain different variants of the 35S and 5S ribosomal RNA gene units. Front. Plant Sci. 13:819750. doi: 10.3389/fpls.2022.819750
93	plant	*Landoltia punctata*	IGS (5S)	molecular methods(cloning, Sanger sequencing, qPCR)	/	-/-	Chen G, Stepanenko A, Borisjuk N (2021) Mosaic arrangement of the 5S rDNA in the aquatic plant Landoltia punctata (Lemnaceae). Front. Plant Sci. 12:678689. doi: 10.3389/fpls.2021.678689
94	plant	*Erysimum*	ITS	Illumina MiSeq sequencing	/	-/-	Osuna-Mascaro C, Rubio de Casas R, Berbel M, et al. (2022) Lack of ITS sequence homogenization in congeneric plant species with different ploidy levels. bioRxiv preprint doi: https://doi.org/10.1101/2022.05.29.493735
95	plant	*Avena bruhnsiana*	ITS1	Next generation sequencing	2/-	-/-	Gnutikov AA, Nosov NN, Loskutov IG, et al. (2022)New insights into the genomic structure of the oats (Avena L., Poaceae): intragenomic polymorphism of ITS1 sequences of rare endemic species Avena bruhnsiana Gruner and its relationship to other species with C-genomes. Euphytica, 218:3

96	plant	*Quercus*	IGS (5S)	high-throughput sequencing amplicon	2/-	-/-	Piredda R, Grimm GW, Schulze ED, et al. (2021)High-throughput sequencing of 5S-IGS in oaks: Exploring intragenomic variation and algorithms to recognize target species in pure and mixed samples. Molecular Ecology Resources, https://doi.org/10.1111/1755-0998.13264
97	plant	*Cucurbita moschata*	IGS (35S)	PacBio sequencing, cloning, Sanger sequencing, FISH	2-12/2	-/-	Matyasek R, Kuderova A, Kutilkova E, et al. (2019) Intragenomic heterogeneity of intergenic ribosomal DNA spacers in Cucurbita moschata is determined by DNA minisatellites with variable potential to form non-canonical DNA conformations. DNA Research 26(3):273-286
98	plant	*Ephedra altissima*	IGS (35S)		14-16/14-16	~6000/~15000	Wang W, Wan T, Becher H, et al. (2019) Remarkable variation of ribosomal DNA organization and copy number in gnetophytes, a distinct lineage of gymnosperms. Annals of Botany, 123:767-781
99	plant	*Gnetum montanum*	35S, 5S	Illumina sequencing, cloning, Sanger sequencing, FISH, Southern-blot	6-8/>10	4000/>100000	Wang W, Wan T, Becher H, et al. (2019) Remarkable variation of ribosomal DNA organization and copy number in gnetophytes, a distinct lineage of gymnosperms. Annals of Botany, 123:767-781
100	plant	*Avena*	ITS	sequence-tagged Roche 454 platform	2-4/-	-/-	Rodionov AV, Krainova L, Gnutikov AA, et al. (2019)Intragenomic polymorphism of internal transcribed spacer ITS1in the locus 35S rRNA of polyploid Avena species. Plant Genetics, Genomics, Bioinformatics, and Biotechnology (PlantGen2019).166-166, DOI: 10.18699/PlantGen2019-148

101	plant	*Camellia*	35S	PCR, Illumina MiSeq sequencing	-/-	-/-	Shao Y, Zhang M, Xu Y, et al. (2018) An improved metagenomic strategy reveals an unprecedentedly high level of intragenomic polymorphism of ribosomal DNA in three species of Camellia. Journal of Systematics and Evolution 56(3):250-258.
102	plant	*Dendrobium*	ITS2	454 pyrosequencing	4-6/2	-/-	Wang XY, Chen XC, Yang P, et ail. (2017) Barcoding the Dendrobium (Orchidaceae) species and analysis of the intragenomic variation based on the internal transcribed spacer 2. BioMed Research International, 2017:2734960
103	plant	*Nicotiana tabacum*		Illumina Hiseq sequencing	8/4	~2000/~2000	Lunerova J, Renny-Byfield S, Matyasek R, (2017) Concerted evolution rapidly eliminates sequence variation in rDNA coding regions but not in intergenic spacers in Nicotiana tabacum allotetraploid. Plant Systematics and Evolution, 3030:1043-1060
104	plant	*Cycas revoluta*	35S	Illumina sequencing, cloning, Sanger sequencing, FISH, Southern-blot	22/2	~17000/-	Wang W, Ma L, Becher H, et al. (2016) Astonishing 35S rDNA diversity in the gymnosperm species Cycas revoluta Thunb. Chromosoma, 125(4):683-699
105	plant	*Panax ginseng*	18S	PCR, cloning, Sanger sequencing	2/-	-/-	Chelomina GN, Rozhkovan KV, Voronova AN, et al. (2016) Variation in the number of nucleoli and incomplete homogenization of 18S ribosomal DNA sequences in leaf cells of the cultivated Oriental ginseng (Panax ginseng Meyer). Journal of Ginseng Research, 40:176-184

106	plant	*Asclepias*	35S	Illumina Hiseq sequencing	-/-	-/-	Weitemier K, Straub SCK, Fishbein M, et al. (2015) Intragenomic polymorphisms among high-copy loci: a genus-wide study of nuclear ribosomal DNA in Asclepias (Apocynaceae). PeerJ 3:e718
107	plant	*Camellia sinensis*	26S	PCR, cloning, Sanger sequencing, FISH	6/-	-/-	Xu J, Xu Y, Yonezawa T, et al. (2015) Polymorphism and evolution of ribosomal DNA in tea (Camellia sinensis, Theaceae). Molecular Phylogenetics and Evolution 89: 63-72
108	plant	*Malus toringoides*	ITS	PCR, cloning, Sanger sequencing	-/-	-/-	Tang L, Tang JM, Tan S, (2015) ITS sequence variation and concerted evolution in the natural hybrid species Malus toringoides. Nordic Journal of Botany 33(1):109–119.
109	plant	*Medicago arborea*	IGS (35S)	PCR, cloning, Sanger sequencing, FISH	2/-	-/-	Galián JA, Rosato M, Rosselló JA. (2014) Incomplete sequence homogenization in 45S rDNA multigene families: intermixed IGS heterogeneity within the single NOR locus of the polyploid species Medicago arborea (Fabaceae). Ann Bot. 114(2):243-51.
110	plant	178 plant species	ITS2	pyrosequencing	-/-	-/-	Song J, Shi L, Li D, et al. (2012) Extensive pyrosequencing reveals frequent intra-genomic variations of internal transcribed spacer regions of nuclear ribosomal DNA. PLoS One.7(8):e43971
111	plant	*Camellia*	ITS	PCR, cloning, Sanger sequencing, FISH	4-6/-	-/-	Vijayan K, Chung MC, Tsou CH, (2012)Dispersion of rDNA loci and its implications on intragenomic variability and phylogenetic studies in Camellia, Scientia Horticulturae .Scientia Horticulturae, 137:59-68

112	plant	*Tortula muralis*	ITS	PCR, cloning, Sanger sequencing	-/-	-/-	Kosnar J, Herbstova M, Kolar F, et al. (2012) A case study of intragenomic ITS variation in bryophytes: Assessment of gene flow and role of polyploidy in the origin of European taxa of the Tortula muralis (Musci:Pottiaceae) complex. Taxon, 61(4):709-720
113	plant	*Byblis liniflora complex*	IGS (5S)	PCR, cloning, Sanger sequencing, FISH	2-4/2-12	-/-	Fukushima K, Imamura K, Nagano K, et al (2011) Contrasting patterns of the 5S and 45S rDNA evolutions in the Byblis liniflora complex (Byblidaceae). J Plant Res.124(2):231-44
114	plant	*Arabidopsis thaliana*	18S	PCR, cloning, Sanger sequencing	4/6	~400/~1000[6]	Incomplete homogenization of 18 S ribosomal DNA coding regions in Arabidopsis thaliana. BMC Research Notes 4(1):93.
115	plant	*Caryophyllaeides fennica*	ITS2	Ag-staining, FISH, PCR, cloning, Sanger sequencing	S, four site/dipl	-/-	Orosová M, Ivica KH, Eva B, et al.(2010) Karyotype, chromosomal characteristics of multiple rDNA clusters and intragenomic variability of ribosomal ITS2 in Caryophyllaeides fennica (Cestoda). Parasitol Int. 59(3):351-357
116	plant	*Cycas*	ITS	PCR, cloning, Sanger sequencing	-/-	-/-	Xiao LQ, Moller M, Zhu H, (2010)High nrDNA ITS polymorphism in the ancient extant seed plant Cycas: Incomplete concerted evolution and the origin of pseudogenes. Molecular Phylogenetics and Evolution 55(1):168-177.
117	plant	*Pyrus*	ITS	PCR, cloning, Sanger sequencing	6/-	-/-	Zheng X, Cai D, Yao L, et al. (2008) Non-concerted ITS evolution, early origin and phylogenetic utility of ITS pseudogenes in Pyrus. Molecular Phylogenetics and Evolution 48: 892-903

118	plant	*Symbiodinium clades A to E*	ITS	PCR, cloning, Sanger sequencing	-/-	-/-	Thornhill DJ, Lajeunesse TC, Santos SR. (2007) Measuring rDNA diversity in eukaryotic microbial systems: how intragenomic variation, pseudogenes, and PCR artifacts confound biodiversity estimates. Mol Ecol. 16(24):532653-40
119	plant	*Rosmarinus officinalis*	ITS	PCR, cloning, Sanger sequencing	-/-	-/-	Rossello JA, Cosin R, Roscaiu M. (2006) Intragenomic diversity and phylogenetic systematics of wild rosemaries (Rosmarinus officinalis L. s.l., Lamiaceae) assessed by nuclear ribosomal DNA sequences (ITS). Plant Systematics and Evolution, 262:1-12
120	plant	*Halophila stipulacea*	ITS	PCR, cloning, Sanger sequencing	-/-	-/-	Ruggiero MV, Procaccini G. (2004) The rDNA ITS region in the lessepsian marine angiosperm Halophila stipulacea (Forssk.) Aschers. (Hydrocharitaceae): intragenomic variability and putative pseudogenic sequences. J Mol Evol. 58(1):115-121
121	plant	*Armeria*	ITS	PCR, cloning, Sanger sequencing	-/-	-/-	Nieto Feliner G, Gutiérrez Larena B, Fuertes Aguilar J. (2004) Fine-scale geographical structure, intra-individual polymorphism and recombination in nuclear ribosomal internal transcribed spacers in Armeria (Plumbaginaceae). Ann Bot. 93(2):189-200
122	plant	*Pinus tabuliformis, P. yunnanensis, P. densata, P. massoniana, P. bungeana*	IGS (5S)	PCR, cloning, Sanger sequencing	/1-2	-/-	Liu ZL, Zhang DM, Wang XQ, et al. (2003) Intragenomic and interspecific 5S rDNA sequence variation in five Asian Pines. American Journal of Botany 90(1):17-24

123	plant	*Leucaena*	ITS	PCR, cloning, Sanger sequencing	-/-	-/-	Hughes CE, Bailey CD, Harris S,(2002) Divergent and reticulate species relationships in Leucaena (Fabaceae) inferred from multiple data sources: Insights into polyploid origins and nrDNA polymorphism. American Journal of Botany 89(7): 1057-1073.
124	plant	*Quercus*	ITS	PCR, cloning, Sanger sequencing	2/-	-/-	Mayol and Rosselló. 2001. Why nuclear ribosomal DNA spacers (ITS) tell different stories in Quercus. Molecular Phylogenetics and Evolution 19.2(2001):167-176.
125	plant	*Aconitum*	ITS	Cloning and PCR-SSCP (single-stranded con formational polymorphisms) analysis	6-20/2-23	-/-	Kita Y, and Ito M. (2000). Nuclear ribosomal ITS sequences and phylogeny in East AsianAconitum subgenusAconitum (Ranunculaceae), with special reference to extensive polymorphism in individual plants. Plant Systematics and Evolution 225(1-4):1-13.
126	plant	*subfamily Calamoideae*	ITS	PCR, cloning, Sanger sequencing	-/-	-/-	Baker WJ, Hedderson TA, Dransfield J. (2000) Molecular phylogenetics of subfamily Calamoideae (Palmae) based on nrDNA ITS and cpDNA rps16 intron sequence data. Molecular Phylogenetics and Evolution 14(2):195-217.
127	plant	*Aeschynanthus*	ITS	PCR, cloning, Sanger sequencing	1-2/	-/-	Denduangboripant J and Cronk QCB (2000) High intraindividual variation in internal transcibed spacer sequences in Aeschynanthus (Gesneriaceae): implications for phylogenetics. Proceedings of the Royal Society B: Biological Sciences 267(1451):1407-1415.

128	plant	*Amelanchier Agamic Complex*		PCR, Sanger sequencing	-/-	-/-	Campbell CS, Wojciechowski MF, Baldwin BG, et al. 1997. Persistent nuclear ribosomal DNA sequence polymorphism in the Amelanchier agamic complex (Rosaceae). Molecular Biology and Evolution 14(1):81-90.
129	plant	*Zea*	ITS	PCR, cloning, Sanger sequencing, restriction enzyme analyses	2/-	-/-	Buckler IV ES, and Holtsford TP (1996) Zea systematics: ribosomal ITS evidence. Molecular Biology and Evolution 13(4):612-22.
130	plant	some species of Winteraceae	ITS1, ITS2	PCR, Sanger sequencing	-/-	-/-	Suh Y, Thien LB, Reeve HE, (1993) Molecular evolution and phylogenetic implications of internal transcribed spacer sequences of ribosomal DNA in Winteraceae, American Journal of Botany, 80(9):1042-1055
131	plant	*Symbiodinium*	ITS2	Pyrosequencing	-/-	-/-	Arif C, Daniels C, Bayer T, et al. (2014) Assessing Symbiodinium diversity in scleractinian corals via next-generation sequencing-based genotyping of the ITS2 rDNA region. Molecular Ecology. 23(17):4418-33
132	plant	*Larix potaninii*	ITS	PCR, cloning, Sanger sequencing	-/-	-/-	Wei XX, Wang XQ, Hong DY, (2003) Marked intragenomic heterogeneity and geographical differentiation of nrDNA ITS in Larix potaninii (Pinaceae). Journal of Molecular Evolution, 57:623-635
133	plant	*Lespedeza*	ITS	Cloning, Sanger sequencing	-/-	-/-	Xu B, Zeng XM, Gao XF, et al. (2017) ITS non-concerted evolution and rampant hybridization in the legume genus Lespedeza (Fabaceae). Scientific Reports 7:40057.

134	plant	*Mammillaria*	ITS	qPCR and Sanger sequencing	2/-	-/-	Harpke D and Peterson A, (2007) Quantitative PCR revealed a minority of ITS copies to be functional in Mammillaria (Cactaceae). International Journal of Plant Sciences 168(8):1157-1160.
135	protist	*Alexandrium tamarense, A. fundyense, A. catenella*	18S	PCR, cloning, Sanger sequencing	2/-	-/-	Miranda LN, Zhuang Y, Zhang H, et al. (2012)Phylogenetic analysis guided by intragenomic SSU rDNA polymorphism refines classification of “Alexandrium tamarense” species complex. Harmful Algae,16:35-48
136	protist	*Dientamoeba fragilis*	ITS1	PCR, cloning, Sanger sequencing	-/-	-/-	Bart A, van der Heijden HM, Greve S,et al.(2008) Intragenomic variation in the internal transcribed spacer 1 region of Dientamoeba fragilis as a molecular epidemiological marker. J Clin Microbiol.46(10):3270-5

Footnotes

[1] 45S is used for animal and fungi units; 35S for plant units

[2] Animal and plant locus number are mostly taken from the Animal and Plant rDNA loci database (https://www.animalrdnadatabase.com and https://www.plantrdnadatabase.com/)

[3] Fungi rDNA copy number are taken mostly from Lofgren et al. (2018), DOI: 10.1111/mec.14995

[4] Values represent the average; considerable interindividual variation in rDNA copy number was reported in humans (Gibbons, J., Branco, A., Yu, S. et al. Ribosomal DNA copy number is coupled with gene expression variation and mitochondrial abundance in humans. Nat Commun 5, 4850 (2014). https://doi.org/10.1038/ncomms5850; Hall, A.N., Turner, T.N. & Queitsch, C. Thousands of high-quality sequencing samples fail to show meaningful correlation between 5S and 45S ribosomal DNA arrays in humans. Sci Rep 11, 449 (2021). https://doi.org/10.1038/s41598-020-80049-y)

[5] Copy number determined from the draft genome (Liu, S., Wang, L., Zheng, H. et al. Comparative genomics reveals Cyclospora cayetanensis possesses coccidia-like metabolism and invasion components but unique surface antigens. BMC Genomics 17, 316 (2016). https://doi.org/10.1186/s12864-016-2632-3)

[6] Variation in 45S rDNA copy number exits between populations of Arabidopsis thaliana in wild (Long, Q., Rabanal, F., Meng, D. et al. Massive genomic variation and strong selection in Arabidopsis thaliana lines from Sweden. Nat Genet 45, 884–890 (2013). https://doi.org/10.1038/ng.2678)

-/-, not determined
