# Supplementary material for: Intragenomic rDNA variation - the product of concerted evolution, mutation, or something in between?
Source: Heredity (Edinb). 2023 Jul 4;131(3):179–88. doi: 10.1038/s41437-023-00634-5 (PMC10462631; doi:10.1038/s41437-023-00634-5)
Supplement: Supplementary file 2 — Supplementary Table S2 [file 41437_2023_634_MOESM2_ESM.pdf]

Table S2: Summarizing information on variability across different subregions of rDNA units

rDNA subregion[1]	Total		Plant		Animal		Fungi		Protist		Prokaryots	
	Number[2]	[%]	Number	[%]	Number	[%]	Number	[%]	Number	[%]	Number	[%]
Coding subregion (16S, 18S, 26S, 28S, 5S)	15	11.0	3	2.2	5	3.7	3	2.2	1	0.7	3	2.2
Non-coding ITS (35S, 45S)	78	57.4	27	19.9	32	23.5	17	12.5	1	0.7	1	0.7
Non-coding IGS (35S, 45S, 5S)	14	10.3	8	5.9	4	2.9	2	1.5	0	0.0	0	0.0
Coding and non-coding subregions[3]	29	21.3	5	3.7	8	5.9	16	11.8	0	0.0	0	0.0
Sum	136	100	43	31.6	49	36.0	38	27.9	2	1.5	4	2.9

[1]The data were collected from Table S1

[2]Number of reports

[3]Studies reporting variation in both coding and non-coding regions and/or whole units
